# Celebrating 350 years of *Philosophical Transactions*: life sciences papers

**DOI:** 10.1098/rstb.2014.0380

**Published:** 2015-04-19

**Authors:** Linda Partridge

**Affiliations:** Editor-in-Chief

This special issue of *Philosophical Transactions of the Royal Society B* has been commissioned to mark the Royal Society's celebration of the 350th anniversary of the publication of the first issue of *Philosophical Transactions*, the world's oldest scientific journal. The issue is designed both to look back at some major, historical, scientific landmarks in the biological sciences published in the journal, and to consider their ramifications for modern understanding and research. A corresponding special issue of *Philosophical Transactions A*, covering the physical, mathematical and engineering sciences, has also been produced [1].

Some 2 years after its formation, ‘The Royal Society of London for Improving Natural Knowledge’ was granted an addition to its charter by King Charles II, allowing it to publish. On 6 March 1665, the first issue of *Philosophical Transactions* was published under the visionary editorship of Henry Oldenburg, a theologian and polymath from the German Hanseatic town of Bremen, who was also the first Secretary of the Society. The information presented in the first volume [2] was both diverse and fascinating, with a wide range of correspondence on topics including ‘A spot in one of the belts of Jupiter’; ‘An account of micrographia, or the physiological descriptions of minute bodies, made by magnifying glasses’ and ‘Of the mineral of Liege, yielding both brimstone and vitriol, and the way of extracting them out of it, used at Liege’. Thus was the continuing, wide, scientific range of the journal established.^1^

The journal has changed considerably in its 350 years, although important features of modern scientific publishing were present from the beginning. At the outset, the journal focused on the latest scientific discoveries and development, and often reported on current, rather than on finished, research. Accurate recording of empirical observations and experimental measurements was paramount, and regarded as the route to understanding the true nature of things. The importance of the archival function of scientific journals became painfully apparent with the Great Fire of London in 1666, when the prospect of all knowledge being lost must have seemed all too real. Indeed, all the remaining stock of *Philosophical Transactions* from that year was destroyed, although thankfully copies still survived outside London. Samuel Pepys, diarist and later President of the Royal Society, rushed to save his own papers from the fire, for there were often no second copies of the notes and correspondence that had acted as a record and established priority on ideas and observations in the days before scientific publishing. The journal eventually became an important source of revenue for the Society, although its finances have at many times since been precarious, and the editorship in those hardship times required an unusual level of devotion to the cause. Appropriate acknowledgement of scientific precedence was also a requirement for publication, and Oldenberg instituted the practice of sending submitted manuscripts to other experts for commentary and feedback to authors, helping to establish today's practice of peer review.

The Royal Society itself underwent something of a dip in the first half of the eighteenth century, when both it and *Philosophical Transactions*, then still officially an independent publication, were subject to biting satires from writers such as Jonathan Swift. The journal was brought under direct Royal Society control in 1752, and renamed as the *Philosophical Transactions of the Royal Society* in 1786. Further reform followed in the nineteenth century, when Charles Babbage wrote an open letter, denouncing fellows who used the Royal Society as a social club, and calling for a new category of fellow: working scientists who published their work in *Philosophical Transactions.* By the late 1830s, peer review was tightened, usually involving two or more referees, who could include the Editor, a position then still held by the Secretary of the Society.[Fig RSTB20140380F1]

**Figure 1. RSTB20140380F1:**
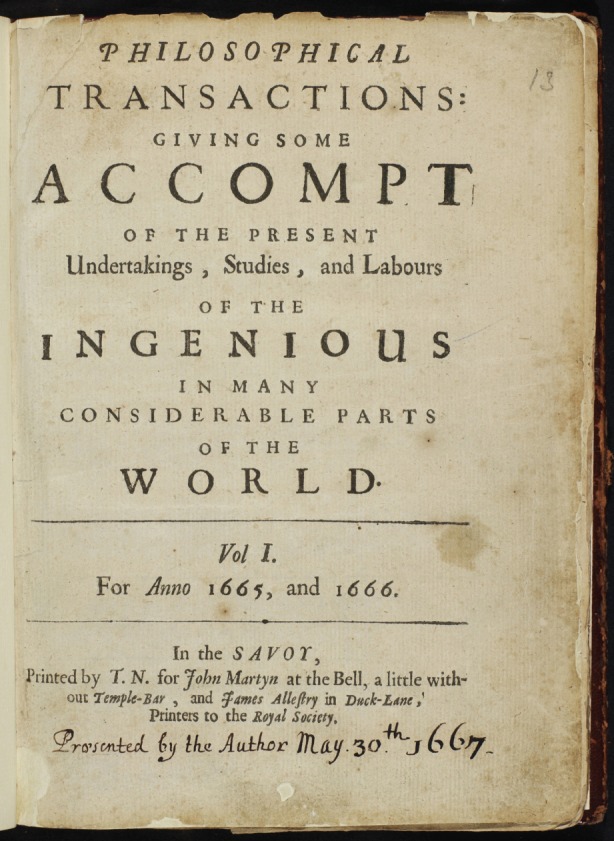
An image of the title page of the first volume of *Philosophical Transactions*. (Online version in colour.)

By 1887, the breadth and volume of science had grown so much that the journal split into two—*Philosophical Transactions A*, covering the physical sciences and *Philosophical Transactions B*, covering the biological sciences. In the late twentieth century, much of the administrative and marketing work for the journals was taken on by in-house publishing editors, with a fellow apiece at the helms of *Philosophical Transactions A* and *B*, supported by large, international and diverse editorial boards of fellows and other scientific experts. The modern *Philosophical Transactions B* also provides a uniquely strong context for the papers that it publishes, by grouping them into themed volumes, on topics of current scientific opportunity and rapid progress and of policy importance, sometimes based on the talks given at Royal Society discussion meetings.

Each commentary in this issue considers a key scientific discovery in biological sciences that was published in *Philosophical Transactions* in the years since 1665 (physical sciences papers are covered in the corresponding issue of *Philosophical Transactions A* [1]). We invited authors who are authorities in the area represented in each earlier publication both to outline the significance of the original paper in the context of the state of knowledge at the time that it was published, and to give an account of how the discovery presented has been developed by subsequent research.

Selecting the papers to include in the volume was not an easy task, because we were confronted with a plethora of strong contenders. We wanted to ensure a reasonable historical spread, and we strove to balance different research areas, which did result in the exclusion of some classic publications in well-represented areas. There is also a woeful lack of publications by female scientists, mainly a sign of the times.

The landscape of scientific publishing has changed rapidly in recent years and continues to do so. As *Philosophical Transactions B* journeys through these latest developments, both we who have the honour to edit the journal, and the powerful teams of scientists and publishers with whom we work, will strive to ensure that the future of the journal is even more remarkable and successful than its past.

## Endnote

1 The Royal Society booklet ‘*Philosophical Transactions: 350 years of publishing at the Royal Society* (*1665–2015*)’ (available at https://royalsociety.org/~/media/publishing350/publishing350-exhibition-catalogue.pdf) provides an account of the origins of the *Philosophical Transactions* and its evolution, together with some biographical details of the key individuals involved.
